# Influence of Surfactant-Mediated Interparticle Contacts on the Mechanical Stability of Supraparticles

**DOI:** 10.1021/acs.jpcc.1c06839

**Published:** 2021-10-18

**Authors:** Junwei Wang, Eunsoo Kang, Umair Sultan, Benoit Merle, Alexandra Inayat, Bartlomiej Graczykowski, George Fytas, Nicolas Vogel

**Affiliations:** † Institute of Particle Technology, Friedrich-Alexander University Erlangen-Nürnberg, Cauerstrasse 4, 91058 Erlangen, Germany; ‡ Max Planck Institute for Polymer Research, Ackermannweg 10, 55128 Mainz, Germany; § Institute of Chemical Reaction Engineering, Friedrich-Alexander University Erlangen-Nürnberg, Egerlandstrasse 3, 91058 Erlangen, Germany; ∥ Materials Science and Engineering I and Interdisciplinary Center for Nanostructured Films (IZNF), Friedrich-Alexander University Erlangen-Nürnberg, 91058 Erlangen, Germany; ⊥ Faculty of Physics, Adam Mickiewicz University, Uniwersytetu Poznanskiego 2, Poznan 61-614, Poland

## Abstract

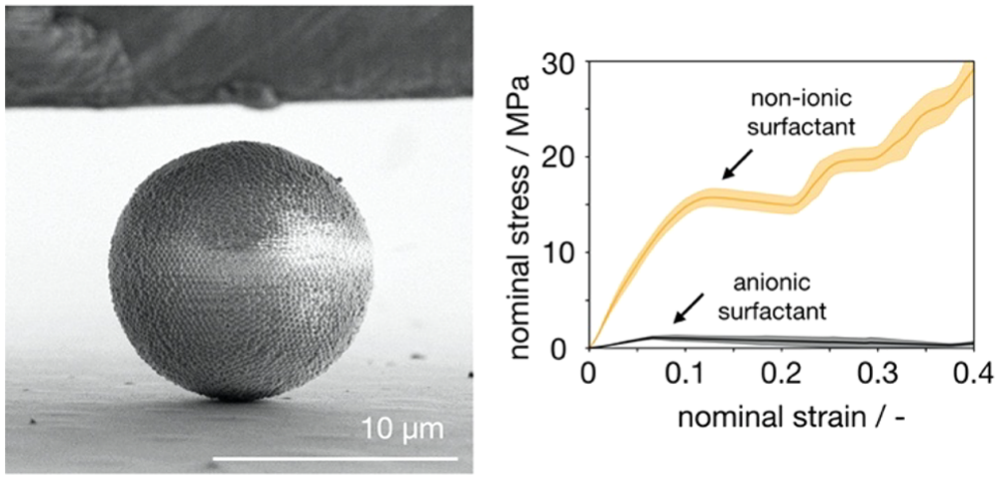

Colloidal supraparticles are micron-scale spherical assemblies of uniform primary particles, which exhibit emergent properties of a colloidal crystal, yet exist as a dispersible powder. A prerequisite to utilize these emergent functionalities is that the supraparticles maintain their mechanical integrity upon the mechanical impacts that are likely to occur during processing. Understanding how the internal structure relates to the resultant mechanical properties of a supraparticle is therefore of general interest. Here, we take the example of supraparticles templated from water/fluorinated oil emulsions in droplet-based microfluidics and explore the effect of surfactants on their mechanical properties. Stable emulsions can be generated by nonionic block copolymers consisting of a hydrophilic and fluorophilic block and anionic fluorosurfactants widely available under the brand name Krytox. The supraparticles formed in the presence of both types of surfactants appear structurally similar, but differ greatly in their mechanical properties. While the nonionic surfactant induces superior mechanical stability and ductile fracture behavior, the anionic Krytox surfactant leads to weak supraparticles with brittle fracture. We complement this macroscopic picture with Brillouin light spectroscopy that is very sensitive to the interparticle contacts for subnanometer-thick adsorbed layers atop of the nanoparticle. While the anionic Krytox does not significantly affect the interparticle bonds, the amphiphilic nonionic surfactant drastically strengthens these bonds to the point that individual particle vibrations are not resolved in the experimental spectrum. Our results demonstrate that seemingly subtle changes in the physicochemical properties of supraparticles can drastically impact the resultant mechanical properties.

## 1 Introduction

Supraparticles are finite assemblies of primary colloidal particles with micron-scale dimensions fabricated by confined self-assembly processes, for example, within emulsion droplets.^1−4^ If the primary particles are sufficiently monodispersed, such supraparticles exhibit a well-defined structural order determined by the interplay between the curvature imposed by the templating droplet,^5−7^ the system size,^8,9^ and the shape of the primary particle,^10−12^ as well as the drying kinetics.^13,14^ As inherently hierarchical materials, supraparticles bridge the microscopic and macroscopic scales and combine emergent properties arising from the collective behavior of the primary building blocks with characteristics of a macroscopic powder and finite properties that can be different from self-assembled materials in the bulk. The size of the primary colloidal particles can be chosen to match the wavelengths of visible light,^15,16^ in which case the defined structural arrangement in the supraparticles produces tunable structural color with an isotropic or anisotropic appearance.^17−19^ The latter depends on whether their internal structure is amorphous,^20^ partially crystalline,^21−23^ or completely crystalline.^24^ Such supraparticles already find use in structural pigments,^25^ displays, and sensors.^26^

The surface topography and composition of supraparticles can be controlled via mixtures of different primary particle sizes and materials, creating hierarchical porous^27^ materials with gradients^13^ for catalysis^28^ and tissue engineering^29^ or powders with enhanced flowability for additive manufacturing.^30^ Incorporating functional primary particles encodes additional properties into such supraparticles, such as magnetic actuation,^31^ efficient separation,^32^ object identification,^33^ antitampering,^34^ lasing,^35^ photothermal conversion,^36^ and drug delivery.^37^ Supraparticles also serve as model systems in soft condensed matter, providing insights for fundamental questions on structure formation,^6,7,38,39^ crystallization pathways,^8,40^ and the number–structure–property relationship^9,41^ of confined finite systems.

For any application, a fundamental prerequisite is that the supraparticles must have sufficient mechanical stability to ensure structural integrity. This is especially important since the emergent properties arise from the defined internal arrangement of the primary particles and will therefore be lost upon breakage or disintegration. The mechanical properties of supraparticles, especially under compression, which is likely to occur in applications, need to take into account both the properties of primary particles and the strength of the interparticle bonds holding these particles together.

On the primary particle level, nanomechanical studies of submicron particles made from polystyrene,^42^ silica,^43^ gold,^44^ or titania^45^ provided detailed mechanical properties such as their Young’s moduli, yield strengths, or elastic-plastic loading indexes based on theoretical Hertz, Johnson–Kendell–Roberts (JKR), or Derjaguin–Muller–Toporov (DMT) models.^46^ At the supraparticle level, pioneering work in the chemical engineering community has built the foundation to understand the breakage^47−49^ and elastic-plastic properties^50−52^ of supraparticles assisted by simulation methods.^53−55^ However, typically produced in large-scale processes such as spray drying,^14^ emulsification,^56^ or wet granulation, the supraparticles in these investigations were inhomogeneous in size and consisted of ill-defined primary particles without a uniform shape and size, rendering a detailed correlation of their structure with the resultant mechanical properties challenging. With advancements in colloidal science, supraparticles with a well-defined internal structure, accurate spherical shape, and narrow size distributions can be fabricated, often by the consolidation of well-defined colloidal primary particles encapsulated in uniform emulsion droplets produced in microfluidics.^5,8,9,15,24,57−59^ The precise control of the size and number of primary particles and, hence, the size and geometric feature of the supraparticles enables a detailed investigation of their size- and structure-dependent mechanical properties.^60,61^

From bulk studies of colloidal assemblies, it has been established that surprisingly attractive mechanical properties can result from a strengthening of the interparticle bonds, for example, by the addition of binders,^48^ sintering,^62^ or chemically interconnecting organic ligands on the surface of the materials.^63−66^ Recently, we focused on the mechanical properties of supraparticles in the absence of such binders. Intuitively, it would be anticipated that such a material easily breaks upon compression due to the relatively weak interparticle contacts.^61^ However, supraparticles made from polystyrene primary particles exhibit a surprisingly high deformation resistance and ductile fracture behavior, which was comparable to solid polystyrene microparticles at ambient conditions.^61^ Based on an adapted Griffith fracture model, we established scaling relationships connecting fracture stress and strain to the dimensions of primary particles and supraparticles. In this context, the interplay between the physical interparticle bonds and the mechanical strength of the individual primary particles determines the complex fracture behavior of supraparticles.

The microfluidic fabrication of such defined supraparticles occurs by evaporating a droplet of aqueous colloidal dispersion in an continuous oil phase. Since no additive is added in the water phase, in the first approximation, the formed supraparticles should be considered a binderless granulate consisting only of the primary particles, held together by van der Waals forces and capillary bridges. However, the fabrication process necessitates the presence of a surfactant that stabilizes the individual droplets.^67−69^ These surfactant molecules, albeit used in small concentration in the continuous oil phase, can remain at the contacts between the primary particles and therefore affect the mechanical properties of the supraparticles. Such influences have not been addressed in our previous study on supraparticle mechanics and, in general, can be easily overlooked in the design of experimental systems to prepare supraparticles from emulsion-based approaches.

Here we focus on the effect of these surfactants and show that even trace amounts of the surfactant molecules can significantly change the mechanical behavior of supraparticles. Depending on the nature of the surfactant and its interaction with the primary particles, the supraparticles can exhibit strongly varying mechanical stability, with a fracture behavior changing from ductile to brittle. We investigate the fracture mechanics of the supraparticles with a detailed study of the interparticle contacts with the different surfactant molecules. To shed light on changes in the interparticle contacts by the presence of surfactants, we employ the noncontact, noninvasive, and zero-strain Brillouin Light Spectroscopy (BLS)^70−76^ to record the primary particle mechanical vibrations in the supraparticles. The vibration spectrum is highly sensitive to the interparticle contacts^77−80^ and thus allows correlating the mechanical properties of the supraparticles under compression with the micromechanics of the interparticle contacts. This correlation helps to rationalize the changes observed in the supraparticle mechanical stability and showcases the possibility of mechanical property design for particulate materials via surfactants.

## 2 Methods

### 2.1 Microfluidic Chip Fabrication

PDMS microfluidic devices were produced by a typical soft lithography method, as described in the literature.^81^ In short, a silicon wafer was spin-coated with SU-8 negative photoresist, and a pattern mask was used to create microstructures on the coated water surface through UV light. The flow-focusing microfluidic channel microstructures were then hardened and coated with an antisticking layer (trichloro-(1*H*,1*H*,2*H*,2*H*-perfluorooctyl)silane, Sigma-Aldrich) to produce the master wafer. Polydimethylsiloxane (Sylgard 184 PDMS from Dow Corning) was mixed with a curing agent at a 10:1 weight ratio and degassed before pouring onto the master wafer to an approximately 1 cm thickness. The PDMS was cured at 80 °C in an oven overnight. The cured PDMS chip was cut off by a razor blade and peeled from the wafer. A biopsy punch (1.0 mm in diameter, Kai Group) was used to create the inlets and outlets in the PDMS chip. The remaining debris and dust on the PDMS chip surface were removed by Scotch adhesive tape. The chip was washed with ethanol and water (Milli-Q) and dried with compressed air. After cleaning, the PDMS chip and a clean glass slide were plasma-treated for 30 s in an oxygen environment at 30 W power (Diener electronic, Femto). After surface activation, the PDMS chip was bonded to the glass slide and put in the oven at 80 °C for 30 min to enhance bonding. Afterward, the microfluidics channels were immediately flushed with 2 wt % fluorosilane dissolved in HFE 7500 oil (3M) by injection through a 1 mL syringe of a blunt needle. After 1 h, the channels were flushed by compressed gas to remove the liquid and kept in the oven to dry.

### 2.2 Supraparticle Fabrication

Styrene, acrylic acid, and ammonium peroxodisulfate were purchased from Sigma-Aldrich and used as received. Polystyrene colloidal particles were synthesized using acrylic acid as comonomer and ammonium peroxodisulfate as initiator in surfactant-free emulsion polymerization following literature protocols.^82^ The resultant aqueous polystyrene colloidal particle dispersion (1 wt %) was used as a dispersed phase in the flow-focusing microfluidic channel. Fluorinated oil HFE 7500 (3M) containing 0.1 wt % of fluorosurfactants was used as the continuous phase. Anionic surfactant, perfluoropolyether carboxylic acid (Krytox 157 FSH, DuPont,) was used as received. Nonionic PFPE-PEG-PFPE surfactant (perfluoropolypropylene glycol-*block*-poly(propylene glycol)-*block*-poly(ethylene glycol)-*block*-poly(propylene glycol)-perfluoropolypropylene glycol-*block*) was synthesized following literature protocols.^68^ The anionic surfactant at the chosen concentration yielded an interfacial tension of approximately 20 mN/m, the nonionic surfactant further reduced the interfacial tension to ∼10 mN/m. The liquids were pumped by a precision pump (Cronus) into the microfluidic channel via medical grade PE tubes at flow rates of 200 and 500 μL/h for the disperse phase and the continuous phase, respectively. The emulsion droplets were collected in a 1.5 mL glass vial for storage and drying. Typically, 20 μL of the emulsion was collected in 200 μL of oil. For spherical supraparticles, the glass vial was stored at room temperature without a closing cap, which allows water to be removed overnight by evaporation and diffusion through the oil phase and thus yields consolidated supraparticles suspended in oil. For crystalline supraparticles, the glass vial was sealed with parafilm in which one hole was poked with a 0.4 mm needle and stored at 5 °C to slow down the evaporation. The drying process usually takes 4 weeks.^9^ These supraparticles were deposited onto a solid substrate for scanning electron microscope characterization (Zeiss GeminiSEM 500), mechanical testing, and Brillouin light spectroscopy.

### 2.3 Compression of Supraparticles via Nanoindentation

Nanoindentation on the colloidal supraparticles was performed in a Nanoindenter G200 (KLA, U.S.A.) with 0.01 nm depth resolution and 50 nN load resolution in displacement-controlled mode. The diamond flat punch indenter had a diameter of 90 μm (Synton MDP). The diameters of the colloidal supraparticles were measured in SEM. For the indentation measurements, the samples were deposited onto a piece of silicon wafer directly from the fluorinated oil phase. The silicon wafer was glued to the sample holder with a minimal amount of superglue (Ultra gel, Pattex), and the samples were left for 24 h for the glue to cure. After mounting the sample holder into the indenter, at least 1 h was allowed for thermal stabilization before starting the compression measurements. The measurements were performed at ambient room condition of 50% relative humidity. The loading and unloading speeds were fixed at 50 nm/s, the maximum displacement set at 60% nominal strain, and the peak holding time to 10 s. A double-sided tape was attached next to the sample holder to facilitate the cleaning of the indenter tip. To this purpose, multiple indentations were performed into the tape after each particle measurement in order to remove the debris sticking to the tip.^61^

### 2.4 Brillouin Light Spectroscopy (BLS)

BLS is an optical technique based on the scattering of light by thermally excited hypersonic (GHz) phonons existing in all materials. For isotropic transparent material, a single acoustic phonon at frequency ω (=2π*f*) is probed at a given wave vector, ±***q*** = ***k***_**i**_ – ***k***_**s**_ selected by the scattering geometry, where ±***q*** is for Stokes and anti-Stokes components, ***k***_**i**_ and ***k***_**s**_ are wavevectors of the incident and scattered light. Using energy and momentum conservation laws, a doublet with frequency, ω = ±*cq*, relative to the central Rayleigh line (ω = 0) appears in the spectrum of the scattered light analyzed by a six-pass tandem-Fabry–Perot interferometer.^66,83^ Here, *c* denotes the sound velocity (longitudinal or transverse) in the medium, and the momentum magnitude *q* depends on the scattering geometry. In the so-called transmission geometry, *q*_trans_ = (4π/λ)sin α, where α = θ/2, with θ being the scattering angle between ***k***_**i**_ and ***k***_**s**_ and λ (=532 nm) is the laser wavelength in vacuum. In a general case, *q* = (4π*n*/λ) sin(θ/2) depends on the material refractive index, *n*.^84^ For localized phonons in space, for example, particle resonances, the frequencies are *q*-independent, as revealed by recording the BLS spectra at two different magnitudes of ***q***.

## 3 Results and Discussion

### 3.1 Colloidal Supraparticles Formed in Emulsion Droplets Stabilized by Surfactants

Colloidal supraparticles with a well-defined internal structure, spherical shape, and low size polydispersity are fabricated using emulsion droplets prepared by droplet-based microfluidics as confinements.^9,21^ In the course of the self-assembled process, water is removed from the droplet by evaporation and diffusion into the oil phase until the colloidal primary particles eventually consolidate into compact supraparticles. The diameter of the supraparticles as well as the material, number, and diameter of the primary particles can be controlled via the droplet size and the concentrations of colloidal primary particles.^9,21^ The kinetics of the drying process determine the internal structure of the supraparticles. At very slow drying conditions, crystalline supraparticles with defined geometrical features are formed. Depending on the system size, either truncated icosahedra, decahedra, or octahedra are formed as minimum energy structures.^8,9,24^ A shorter drying time yields spherical supraparticles. These structures exhibit a well-ordered arrangement with close-packed particle layers at the surface and a more disordered inside^21^ and are the most commonly observed type of colloidal supraparticles.^15,85^

**Figure 1:**
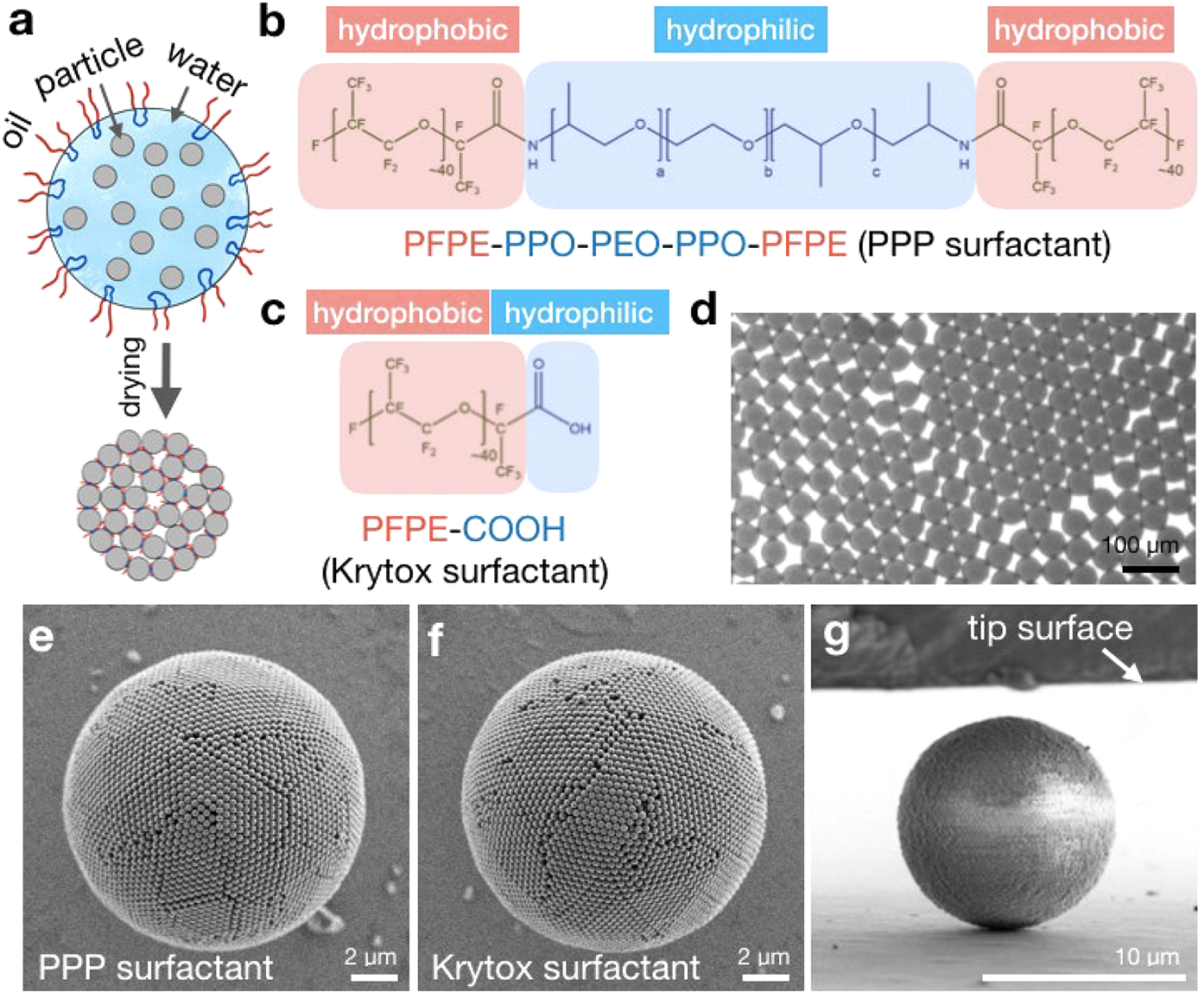
Emulsion-templated fabrication of supraparticles. (a) Schematic illustration of the supraparticle self-assembly in an emulsion droplet and the role of surfactants. (b) Nonionic perfluoropolyether-*block*- (polyethylene glycol-*block*-polypropylene glycol-*block*-polyethylene glycol)-*block* perfluoropolyether surfactant (PPP) used in supraparticle fabrication. (c) Anionic Krytox surfactant used in supraparticle fabrication. (d) Monodispersed water in oil droplets (gray) encapsulating polystyrene colloidal particles produced by the microfluidic device. (e, f) Colloidal supraparticles with spherical shape and ordered surface structure, made with PPP and Krytox surfactant, respectively. (g) Colloidal supraparticle under a nanoindenter tip.

As suggested from Figure 1a, the stability of the emulsion droplets during formation and self-assembly of primary particles depends on the presence of surfactant molecules to stabilize the droplet interface. In this study, we use a water/fluorinated oil system that is typically used in PDMS-based microfluidic devices because the fluorinated oil does not swell the PDMS and therefore ensures reliable operation.^86^ We use two types of surfactant that efficiently stabilize the water/fluorinated oil interface and are often used in such microfluidic systems.^67,68^ The nonionic surfactant, illustrated in Figure 1b, is a triblock copolymer formed by a water-soluble middle part and two perfluoropolyether polymers to induce steric repulsion in the continuous fluorinated oil phase. The hydrophilic block (blue) itself is based on a PPO-PEO-PPO triblock copolymer (polyethylene glycol-*block*-polypropylene glycol-*block*-polyethylene glycol) with a molecular weight MW = 900 g/mol and is commercially available under the trade name of “Jeffamine”. The perfluoropolyether is based on a commercially available fluorinated oil under the name Krytox (Krytox 157 FSH, MW = 7000 g/mol). These Krytox molecules can also directly function as an anionic surfactant. As illustrated in Figure 1c, Krytox is a perfluoropolyether with a carboxylic acid termination, which is deprotonated in an aqueous environment and thus negatively charged. Note that while conventional charged surfactants stabilize emulsions via electrostatic repulsion, in this case, the charged moiety sits in the dispersed phase, while the surfactant exposes the fluorophilic polymer to the continuous phase and therefore induces steric stabilization.

At a concentration as low as 0.1 wt %, both surfactants can efficiently stabilize the water-in-fluorinated oil emulsion (Figure 1d). The uniformity of the droplet results in supraparticles with near-monodispersed sizes, which allows careful investigation of their mechanical properties. Both surfactants repel the negatively charged polystyrene particles (PPP surfactant by steric and Krytox by electrostatic repulsion) from the droplet interface and allow the formation of supraparticles with spherical outline and ordered surface without discernible structural differences (Figure 1e,f). However, as we will see, the mechanical stability of both types of supraparticles is heavily influenced by the type of the surfactants.

### 3.2 Supraparticles with High Mechanical Stability

We first demonstrate that supraparticles formed with nonionic surfactant (abbreviated as “PPP”) exhibit high mechanical stability under compression. We use supraparticles with a diameter of 7 μm consisting of 244 nm polystyrene primary particles. We use an indentation tip of 90 μm to exclude any edge effects and ensure that the supraparticle is compressed between two parallel plates. The force and displacement of the indenter tip is recorded from first contact to the supraparticle until the tip advances up to a nominal strain of 60% (i.e., 4 μm into the sample) during deformation. Since the diameter of the supraparticle during compression changes dynamically during the compression experiment, we define the nominal stress by normalizing the force by the initial supraparticle cross-sectional area and we define the nominal strain by normalizing the displacement by the initial supraparticle diameter. We define the slope in the linear region of the nominal stress–strain curve before fracture as the deformation resistance of the supraparticle. We define the first pop-in (or jump in displacement) in the nominal stress–strain curve as the first fracture event, and the area under the curve up to fracture as the work of deformation.^61^

**Figure 2:**
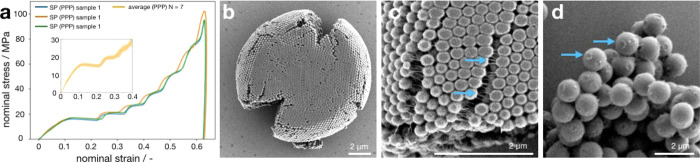
Supraparticles with nonionic PPP surfactant exhibit ductile mechanical properties and high stability from favorable surfactant-particle interactions. (a) Supraparticles formed by polystyrene primary particles and the nonionic PPP surfactant show a reproducible compression behavior with ductile fracture. Multiple, defined plateaus indicate multiple crack formation events without breakage of the supraparticle. (b–d) SEM investigation of the supraparticle surface and morphology. (b) Supraparticle after compression shows several large cracks at the periphery but remains as a single piece. (c) Close up of a deformed supraparticle. Individual primary particles are heavily deformed and solid material bridging neighboring particles is seen. (d) Rig-like residues at the primary particle surface indicate the formation of solid deposits at the contact points between primary particles.

The nominal stress–strain curve of three typical supraparticles is shown in Figure 2a. All three curves show a linear region leading up to a pop-in at about 15 MPa at 10% nominal strain, indicating a first fracture event. Several consecutive plateaus indicate multiple fractures that occur in the course of compression. Noteworthily, these plateaus also demonstrate that the supraparticle deforms in a ductile manner and remains in one piece after fracture. The almost vertical unloading curves suggest predominantly plastic deformation. An average nominal stress–strain curve of seven other samples with the same diameter is shown in the inset by the solid yellow line. The standard deviation is represented by the color band (Figure 2a, inset) and shows the good uniformity of the mechanical properties, which arises from the uniform character of our supraparticles.

In a simplified view, the supraparticles are granular assemblies of primary particles held together only by van der Waals and capillary forces acting between their contact points. For such a system, the supraparticles exhibit a surprisingly high ductility and mechanical stability of 15 MPa fracture stress. We examine the supraparticle morphology before and after compression. Figure 2b shows a deformed supraparticle after compression, exhibiting several large cracks extending from the periphery to the center. We hypothesize that these cracks correspond to the multiple plateaus in the nominal stress–strain curve. Evidently, the supraparticle shows ductile deformation and remains as one single piece after fracture. In Figure 2c, clear deformation of primary particles can be observed. At the top surface, where the supraparticle was in contact with the flat indenter, the spherical primary particles increasingly flatten into a disk shape with increasing diameter and reduced interstitial volume. Importantly, solid bridges (blue arrows) connecting neighboring primary particles are observed within the small cracks, likely resulting from a viscous polymer material residing at the contact points between the primary particles. In Figure 2d, a similar solid residue is evident in a ring-like structure at the surface of primary particles (blue arrows) in a rare broken fragment of a supraparticle after compression (supraparticles consisting of 1 μm primary particles were used to illustrate the residue morphology). The location of these solid features at the surface gives evidence that it was formed in the contact points between neighboring primary particles within the supraparticle. Since the supraparticles are suspended in the fluorinated oil phase before deposition to the solid substrate and mechanical investigations, it can be inferred that the surfactant molecules at least partially infiltrate the porous supraparticle structure. Capillary bridges forming during drying will subsequently drag them toward the contact points where they accumulate. Seemingly, even at the low surfactant concentration of 0.1 wt % used in our fabrication process, the nonionic surfactant molecules precipitate around the contact points of the primary particles inside the supraparticle. Estimation of the dimensions of the formed rings from SEM images gives an approximate volume ratio between the residue surfactant to the particle, which we estimate to be around 0.02%. This value is significantly lower than the typical binder content up to 10% in industrial agglomeration,^87^ yet it effectively stabilizes the supraparticles.

Interestingly, contrary to the common belief that reducing the void volume or increasing the number of contacts increases the mechanical strength of particulate materials,^48,88^ we do not observe an increase of the deformation resistance or fracture stress in crystalline supraparticles,^9,24,41^ which minimizes void volume and maximize contacts (Figure S1a). We distinguish the crystalline supraparticle by its faceted surface, indicating a complete ordered interior structure,^9,24,41^ in contrast to the disordered interior region of spherical supraparticles with only an ordered surface.^21^ In addition, the crystalline supraparticles do not exhibit a tendency to break along crystal planes in the compression experiment at ambient conditions, as may be expected for an atomistic system.^89^ Noteworthily, this behavior changes when the supraparticles are subjected to mechanical stress directly in the oil phase. In this case, the surfactant may reside at the supraparticle surface but is not assumed to infiltrate and manipulate the contact points. A treatment of 30 s ultrasonication to induce mechanical stress in a supraparticle suspension shows that crystalline supraparticles tend to fracture into defined pieces along their (111) crystal planes, while spherical supraparticles with an amorphous core survive without fracture (Figure S1b), retaining the similarity to the expected behavior of atomic materials. These differences in the behavior of crystalline supraparticles further suggest that the mechanical stability in ambient conditions is not entirely controlled by van der Waals and capillary forces, as is the case for typical granular materials, but mediated by the infiltrated surfactants. It also suggests that the PPP surfactant residues may strengthen the contacts, hence, the unexpected ductile deformation of the supraparticles, as we will argue below.

### 3.3 Supraparticles with Low Mechanical Stability

**Figure 3:**
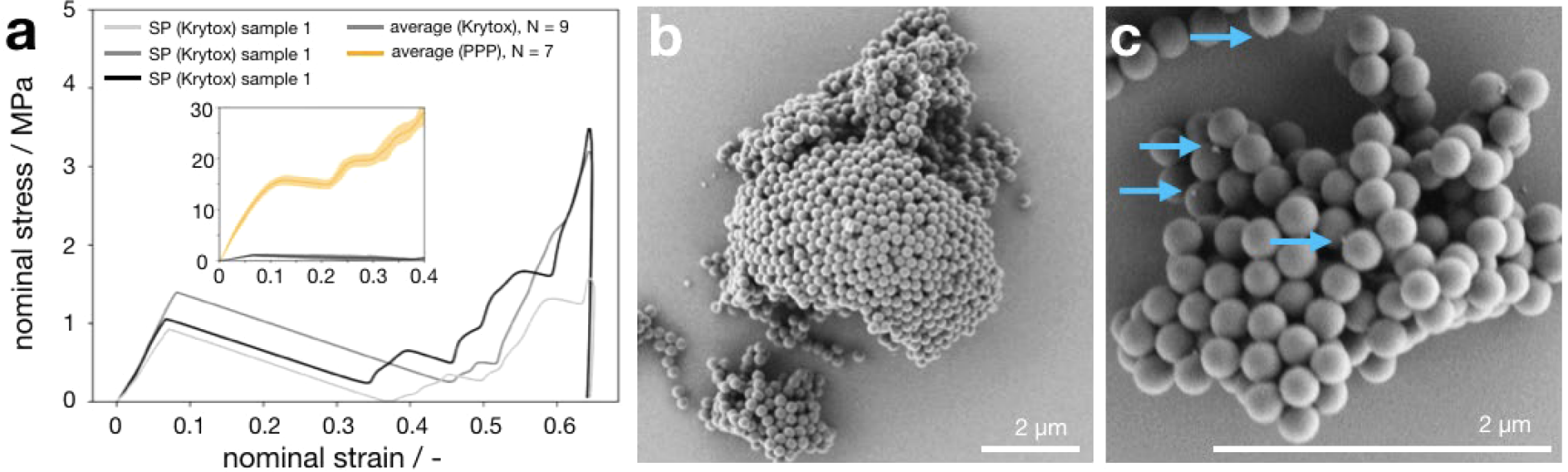
Supraparticles with Krytox surfactant exhibit brittle mechanical properties and low stability from unfavorable surfactant-particle interactions. (a) Supraparticles formed by primary polystyrene particles and the anionic Krytox surfactant show brittle fracture with reduced fracture stress and strain. (b) Supraparticles fracture in a brittle way without visible deformation of the individual primary particles. (c) Surfactant traces reside on primary particle surface as tiny spherical dots.

We change the surfactant from nonionic PPP to anionic Krytox and observe a completely different mechanical behavior. Three individual nominal stress–strain curves for these supraparticles are shown in Figure 3a. Both the supraparticle diameter (7 μm) and the primary PS particle diameter (244 nm) remain the same, but their fracture stress drops significantly from 15 MPa (orange, PPP surfactant, inset) to about 1 MPa (black, Krytox surfactant). In addition, the first fracture occurs at a smaller strain at about 6%, compared to 12% for supraparticles made with PPP surfactant. Noteworthily, after the initiation of a pop-in fracture event, the stress decreases (instead of forming a plateau in the case of PPP surfactant), meaning that the indenter tip experiences hardly any resistance any longer as it advances after the supraparticle fractures. This suggests brittle and catastrophic fracture of the supraparticles where the structure of the deformation-resisting supraparticle changes abruptly and the contact between the indenter and the supraparticle may be reduced or lost. This is confirmed by SEM images of compressed supraparticles (Figure. 3b). Contrary to ductile supraparticles fabricated with nonionic PPP surfactant (Figure 2b), only pieces of supraparticles fabricated in anionic Krytox remain on the substrate. The primary particles remain spherical and undeformed. This, together with the reduced deformation resistance, as well as the ductile to brittle transition, agrees with change of supraparticle mechanical properties induced by weakened interparticle adhesion.^61^ The anionic surfactants do not form bridges that connect neighboring primary particles, as seen in Figure 2c, but are observed to be tiny spherical dots at some primary particle surfaces (Figure 3c), suggesting dewetting from the negatively charged polystyrene surface. Note that despite the observed dewetting from the particle surfaces and contact points of the formed supraparticles, the Krytox surfactant is clearly able to stabilize the water in oil emulsion and allows the formation of supraparticles. The dewetting therefore seems to occur only at the last stages of the drying process.

Table 1 compares the values of typical mechanical properties extracted from the compression curves of supraparticles made with PPP and Krytox surfactant. Although the surfactant residue takes up as little as approximately 0.02% volume, it significantly influences the mechanical behavior of the entire supraparticles.

**Table 1:** Typical Mechanical Properties of Supraparticles

	fracture stress (MPa)	fracture strain (%)	deformation resistance (MPa)	work of deformation (pJ)
with PPP	15	12	125	320
with Krytox	1	6	16	40

### 3.4 Molecular Interpretation

**Figure 4:**
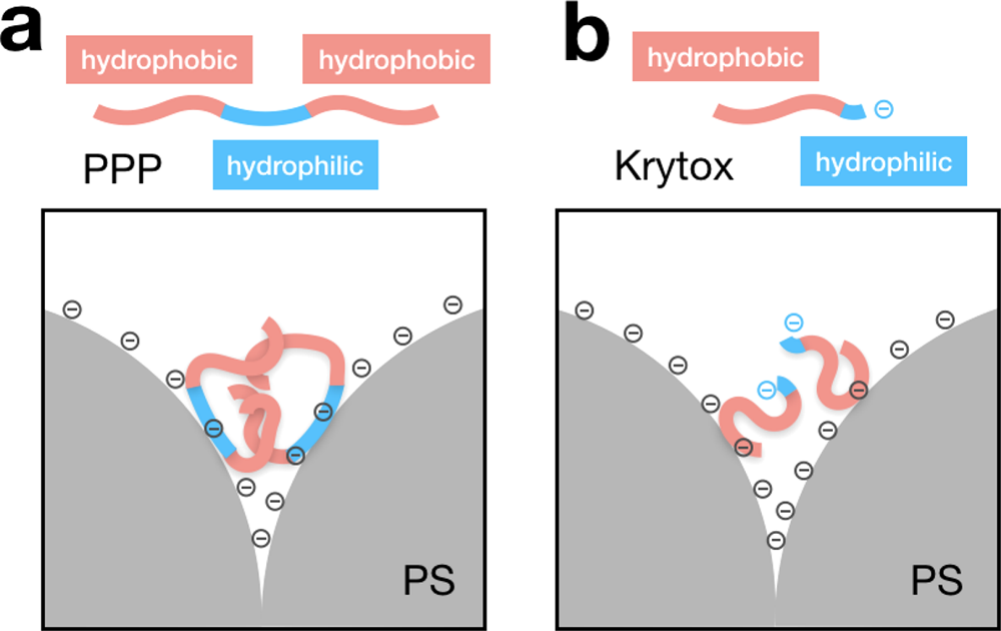
Hypothesis on the influence of surfactant in supraparticle mechanical stability. (a) Nonionic PPP surfactant has a PPO-PEO-PPO block that adsorbs to polystyrene surface due to hydrophobic interactions, the polymer chains in the PFPE blocks from different surfactant molecules are entangled. The PFPE polymer chains are viscous at room temperature and promote adhesion between neighboring particles. (b) Anionic Krytox surfactant has a carboxyl group that deprotonates in ambient humidity to carry a negative charge, which causes an electrostatic repulsion with the negatively charged particle surface. The PFPE block also dewets the particle surface. The surfactant molecule resides at the surface without providing extra adhesive forces. The sketch is not drawn to scale.

**Figure 5:**
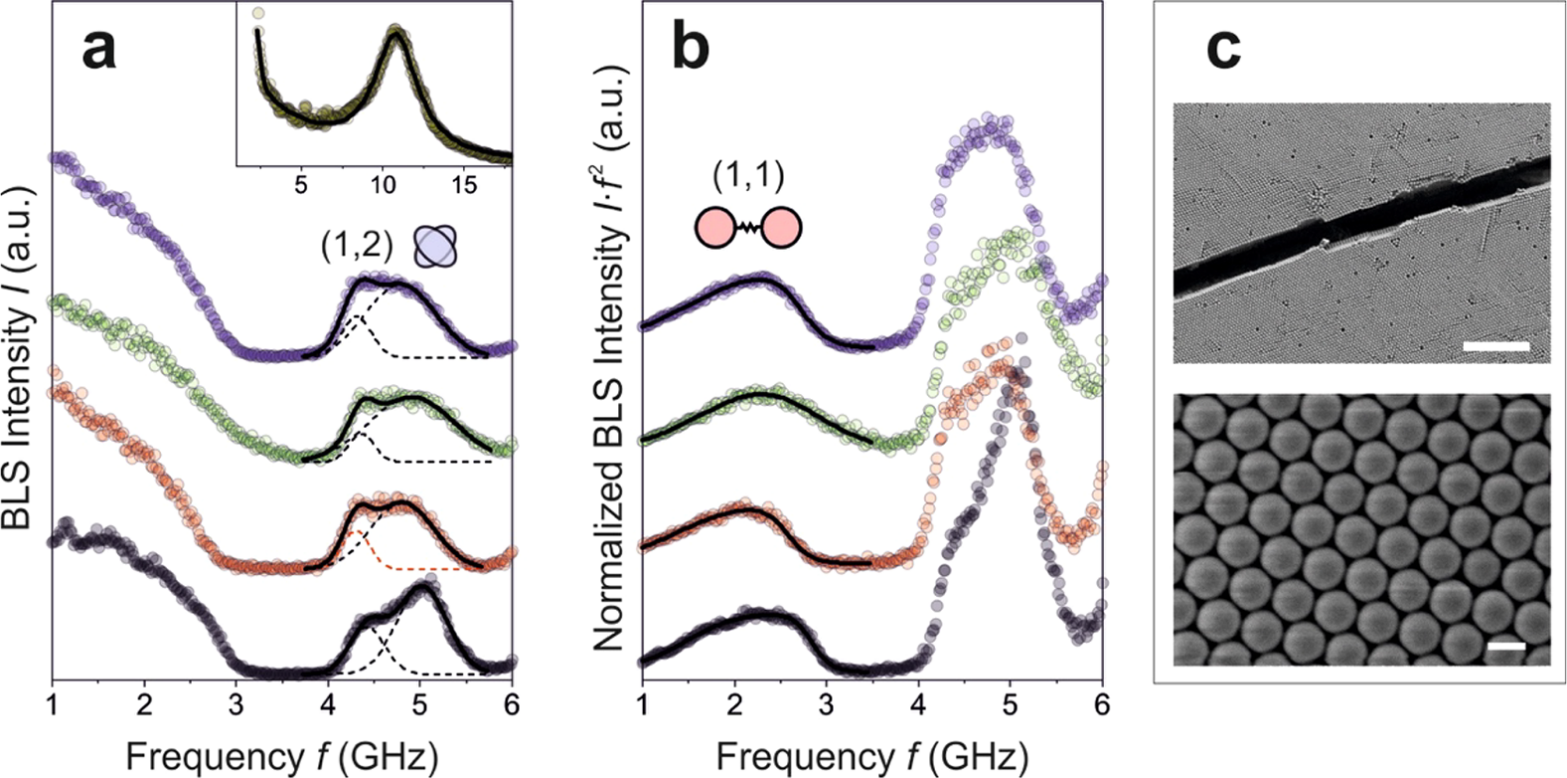
Brillouin light spectroscopy (BLS) spectra (circles) of a colloidal crystal of polystyrene primary particles in (a) normal *I*(*f*) and (b) normalized *I*(*f*)·*f*^2^ presentations. The spectra show the as prepared, dry colloidal crystal film (gray), as well as the film infiltrated with HFE oil containing 0.1% nonionic PPP surfactant (red), pure HFE oil (green), and HFE oil containing 0.1% anionic Krytox surfactant (purple). Dashed lines show for Gaussian line shape fits. Inset to (a): The single peak BLS spectrum recorded in a backscattering geometry shows the same film infiltrated only with PPP. (c) SEM images of the colloidal crystal film as fabricated. Scale bars in (c) are 5 μm (upper) and 200 nm (lower panel).

The contrasting different mechanical properties of the supraparticle containing minute nonionic or anionic surfactant residues suggests a unique effect of the surfactant molecules at the primary particle contacts. As illustrated in Figure 4, we postulate that the different molecular properties of the two types of surfactants modify the adhesion between neighboring primary particles, which results in the widely different behavior of supraparticles. For the nonionic PPP surfactant, the amphiphilic PPO-PEO-PPO block is known to adsorb and cover the polystyrene particle surface.^90−92^ The remaining PFPE blocks form a viscous material, a natural state of this type of polymer at room temperature. Together, these properties allow the surfactant to increase the adhesion between neighboring primary particles (Figure 5a). In addition, the hydrophilic nature of the polymer may be beneficial to bind water and thus strengthen capillary bridges. Separating these two particles requires not only overcoming the van der Waals and capillary forces, but also energy as mechanical work to deform and stretch the viscous polymer surfactant. With PPP surfactant, the mechanical stability of the supraparticles is therefore enhanced. In contrast, the carboxylic head of the Krytox surfactant carries a negative charge and is therefore repelled from the negatively charged particle surface due to the molecular layer of water in ambient conditions (Figure 4b). The PFPE block (which accounts for more than 99% of the surfactant) does not wet the particle surface and hence does not provide any additional adhesive force between neighboring particles. The hydrophobic PFPE polymer may even reduce the formation of capillary water bridges and act as a lubricant between neighboring particles, reducing the mechanical stability of supraparticles. According to this hypothesis, the nonionic PPP surfactant strongly binds neighboring primary particles, connecting all polymeric particles in the supraparticle through polymer bridges into effectively a large porous polymer material. In contrast, the Krytox surfactant does not enhance the adhesion or may even weaken the supraparticles. Visual inspection of broken supraparticles supports this hypothesis. While supraparticles fabricated with the PPP surfactant display large ring-like residues around the contact points (Figure 2d), Krytox surfactant seemingly leaves dot-like residues (Figure 3c) that do not seem to interconnect primary particles.

We use Brillouin light spectroscopy (BLS) to test the hypothesis. To this end, we record the vibration spectrum of both supraparticles and individual primary particles with and without the different surfactants and investigate their effect on the particle contacts via the changes to the characteristic vibrations. In the absence of any interparticle interactions, the individual primary particles exhibit the characteristic spheroidal Lamb (*n*, *l*) modes with *n* and *l* denoting the radial and angular momentum numbers, respectively. In this case, the lowest peak of the vibration spectrum is the (1,2) mode at the frequency *f*(1,2) = *Ac*_t_/*d*, where *c*_t_ is the transverse sound velocity in the primary particle, *d* is diameter, and *A* (≈0.84 for polystyrene) is a constant. The vibration spectrum is very sensitive to interparticle interactions, manifested by the appearance of a new low-frequency mode, termed interaction mode, and a split and concurrently blue shift of the (1,2) mode. The low-frequency mode is assigned to a longitudinal phonon in a cluster of particles. It corresponds to the (1,1) translational mode, which is of zero frequency in independent particles, that is, no particle–particle interaction.^66,83,93,94^ The strength and number of contacts of individual particles in a cluster influence their vibration eigenmodes. Thus, if primary particles in the supraparticles strongly adhere to their neighbors, their individual vibration modes cannot be experimentally resolved. Both the split of the (1,2) mode and the emergence of the interaction induced (1,1) mode allow for a subnanometer thickness sensitivity for adsorbed layers atop of nanoparticles.^95,96^

Figure 5a displays the experimental BLS spectra for a flat colloidal crystal film (black) of the same polystyrene primary particles with *d* = 236 nm, formed by the deposition and ordering of colloidal particles in a drying droplet of colloid dispersion on a solid substrate. We use this colloidal crystal film with a well-ordered arrangement of the primary particles in a face-centered cubic lattice (Figure 5c) as a reference material to probe how the BLS spectra change upon exposure to surfactants that occur in the supraparticle formation. To this end, we first infiltrate the colloidal crystal with the following liquid by dropcasting the liquid into the colloidal crystal: pure HFE oil (green); HFE oil with nonionic 0.1% PPP surfactant (red); HFE oil with 0.1% anionic Krtyox surfactant (purple). A total of 5 μL of the liquid is dropcasted above the center of the colloidal crystal film, which subsequently spreads and is immersed in the film. BLS spectra are recorded after the liquid dried under ambient room conditions, which left the surfactants inside the colloidal crystal film.

The strong wing at about 1–3 GHz indicates the presence of the interaction-induced (1,1) mode. This spectral feature is better resolved in the normalized spectrum *I*(*f*)·*f*^2^ displayed in Figure 5b. Here, the prefactor *f*^2^ accounts for the population of the low frequency thermally excited phonons.^83^ A distinct discernible broad (1,1) peak at about 2.5 GHz is resolved for all infiltrating liquids (pure HFE oil, oil with PPP surfactant, and with Krytox surfactant). Similarly, the particle interactions cause a split of the (1,2) mode at about 5 GHz. The split of the (1,2) mode can be represented by two Gaussian lineshapes (Figure S2) centered at *f*_1_ and *f*_2_ > *f*_1_. The frequency *f*_L_ = 2*f*_1_ – *f*_2_ accounts for the presence of the interparticle interactions, that is, it is equal to the frequency of (1,2) in the case of a single primary particle. While the position, *f*_1_, and *f*_2_ and their relative intensities of the two peaks vary among the different infiltrated polystyrene colloidal crystal films, the frequency, *f*_L_ = 2*f*_1_ – *f*_2_ that accounts for the presence of interparticle interactions *f*_L_ = 3.82 ± 0.07 GHz remains unaffected by the infiltrated fluids. The frequency *f*_L_ (=0.84*c*_t_/*d*) represents the shear modulus, *G* = ρ*c*_t_^2^ (ρ is the mass density) of the polystyrene primary particles. The value of *G* = 1.1 GPa (for *d* = 236 nm) is experimentally the value of bulk polystyrene. The robust vibration spectrum indicates that the vibration spectra of the pure polystyrene colloidal crystal can be recovered upon infiltration with surfactants used in the supraparticle formation. Note, however, that the treatment in both protocols cannot be strictly identical: while a supraparticle is fully surrounded by surfactants, in the case of the flat film, the distribution of surfactant is likely less uniform. To probe how a spectrum of a fully infiltrated colloidal crystal would appear, we added pure PPP surfactant to the colloidal crystal. The resulting spectra shown as an inset to Figure 5a do not show any particle vibrations and only display a peak at about 11 GHz. This spectrum was recorded in a backscattering geometry and corresponds to the effective medium longitudinal sound velocity (∼1900 m/s). In this case, the PPP clearly induces sufficient interparticle adhesion to prevent individual particle vibrations entirely.

**Figure 6:**
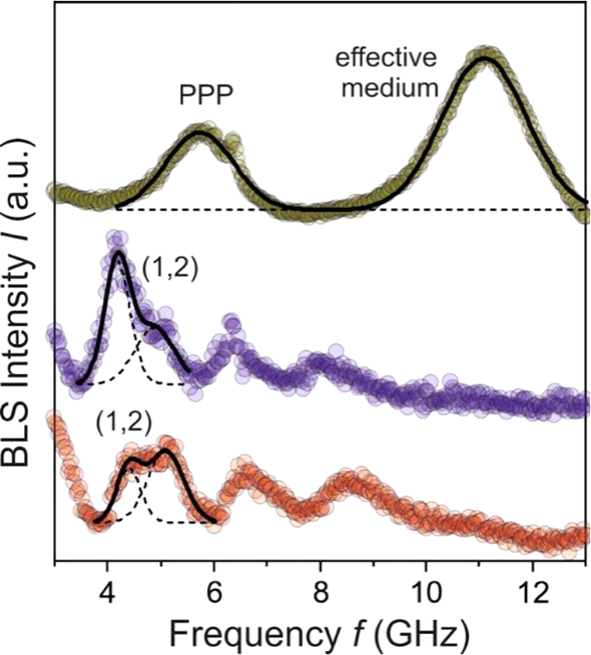
BLS spectra of supraparticles fabricated in two different surfactants. The red and purple curves correspond to crystalline and disordered supraparticles using Krytox surfactant. The interaction-induced split of the (1,2) mode is represented by two Gaussian peaks (dashed lines). The green curve corresponds to the supraparticle using the nonionic PPP surfactant. Notably, no particle vibrations are resolved, and the BLS spectrum shows signatures characteristic of two propagating acoustic phonons.

Next, we focus on the vibration spectra of individual supraparticles prepared from the two different surfactants. To investigate individual supraparticle, we employ micro-BLS that provides a spot size of the probe beam of about 1 μm.^93^Figure 6 displays the BLS spectra of crystalline and disordered supraparticles (Figure S1), both fabricated in HFE oil containing 0.1 wt % of the anionic Krytox surfactant. In this case, elastic vibrations of the constituent primary PS particles are observed. Similar to the flat colloidal crystal film, the single quadrupolar mode (1,2), observed for noninteracting spherical colloidal particles, is split in both ordered and disordered supraparticles, as shown in Figure 6. The two supraparticles reveal minor differences in the (1,2) peak shape characterized by different relative intensities of the two spectral contributions (dashed lines in the lower panel of Figure 6) and *f*_1_, *f*_2_ values. The different packing in crystalline and disordered particles can cause different contacts between the primary polystyrene particles sensitively reflected in the shape of the (1,2) mode split. The comparison of the intensity ratios *f*_2_/*f*_1_ between the crystalline and the disordered supraparticle suggests stronger interactions for the crystalline supraparticle, which is expected due to the larger number of neighbors. Notably, the spectra of the crystalline supraparticle is very similar to the colloidal crystal film, underlining the high degree of crystallinity in this supraparticle (Figure S3). From the computed *f*_L_ (=2*f*_1_ – *f*_2_) it follows that the shear modulus has the same value (1.2 GPa) for the two supraparticles (Figure 6), which agrees with the value of the primary PS particles (Figure 5).

In contrast, the BLS spectrum of a supraparticle fabricated in HFE oil containing 0.1 wt % PPP surfactant drastically changes the BLS spectrum. No individual particle vibrations are resolved in the experimental spectrum. Instead, the BLS spectrum shows longitudinal acoustic phonons propagating in the effective medium. While a single effective medium phonon is observed in the crystalline films of the polystyrene primary particles (inset of Figure 5a), the spectrum of the supraparticle (upper spectrum in Figure 6) displays two acoustic phonons at about 5.8 and 11 GHz. The latter frequency is the same in both systems phonon and represents the effective medium acoustic phonon, whereas the lower frequency mode in PPP coincides with the longitudinal phonon in the neat PPP (Figure S4). The presence of this phonon implies either an inhomogeneous medium with submicron PPP microregions exceeding the phonon wavelength (∼200 nm) or a thin, continuous surface PPP layer. We hypothesize that such pockets may form as residues at the contact points, while a surface layer may form upon drying of the supraparticle as the surfactant stabilizing the shell interaction with the outermost surface of the PS primary particles via hydrophobic interactions of the propylene glycol groups, as discussed in Figure 4 above. The BLS investigation of the interparticle contacts clearly indicates a strong increase in contact strength in the presence of PPP surfactant, corroborating with the increased resistance to mechanical compression shown above. In contrast, using Krytox as a surfactant did not alter the particle vibrations, corroborating with the hypothesis that this type of surfactant does not re-enforce the interparticle contacts. As a consequence, supraparticles prepared with Krytox exhibit lower mechanical properties and break in a brittle manner. Note that while the affinity of the surfactant to the particles enhance their mechanical stability, it needs to be carefully balanced with the primary role of the surfactant, which is to ensure emulsion stability during the supraparticle formation process. In particular, using strong attraction, such as the anionic Krytox surfactant with positively charged polystyrene colloidal particles, we observe that individual primary particles leave the emulsion droplet and thus do not form supraparticles in our emulsion-based process.

## 4 Conclusions

We show that the mechanical stability of colloidal supraparticles is mediated by the inevitable surfactant residue from the emulsion fabrication step. Using the example of a microfluidic production process with perfluorinated oil as the continuous phase, we probe this difference by two types of commonly used surfactants. Even at a trace amount, using the nonionic block copolymer-based surfactant PPP results in mechanically stable supraparticles that exhibit high deformation resistance and ductile fracture behavior. The anionic surfactant Krytox, in contrast, yields supraparticles with mechanical properties that are reduced by about 1 order of magnitude from 15 MPa fracture stress to 1 MPa. In addition, the fracture behavior with this type of surfactant changes from ductile to brittle. We complement the macroscopic mechanical investigation with a microscopic monitoring of the interparticle contacts via Brillouin light spectroscopy. These investigations corroborate the macroscopic interpretation that the nonionic surfactant strongly strengthens the interparticle contacts to the point that no individual vibrations can be resolved. Our work sheds light on the critical role of surfactants in tailoring the mechanical properties of colloidal supraparticles and provides guidelines for the fabrication of mechanically robust supraparticles, which are the prerequisite for any targeted applications of such materials.

## Supporting Information Available

Details of nominal stress–strain curves of crystalline and spherical supraparticles, BLS spectrum of supraparticles and crystalline films, and backscattered spectra of two surfactants (PDF)
