# Supplementary material for: Influence of Surfactant-Mediated Interparticle Contacts on the Mechanical Stability of Supraparticles
Source: J Phys Chem C Nanomater Interfaces. 2021 Oct 18;125(42):23445–56. doi: 10.1021/acs.jpcc.1c06839 (PMC8558861; doi:10.1021/acs.jpcc.1c06839)
Supplement: Supplementary file 1 — jp1c06839_si_001.pdf [file jp1c06839_si_001.pdf]

Supporting information

Influence of Surfactant-Mediated Interparticle Contacts on the Mechanical Stability of Supraparticles

Junwei Wang[1], Eunsoo Kang[2], Umair Sultan[1,3], Benoit Merle[4], Alexandra Inayat[3], Bartlomiej Graczykowski[2,5], George Fytas[2,]*, Nicolas Vogel[1,]*

1 Institute of Particle Technology
Friedrich-Alexander University Erlangen-Nürnberg, Cauerstrasse 4, 91058 Erlangen, Germany

2 Max Planck Institute for Polymer Research
Ackermannweg 10, 55128, Mainz, Germany

3 Institute of Chemical Reaction Engineering
Friedrich-Alexander University Erlangen-Nürnberg, Egerlandstrasse 3, 91058 Erlangen, Germany

4 Materials Science & Engineering I and Interdisciplinary Center for Nanostructured Films (IZNF)
Friedrich-Alexander University Erlangen-Nürnberg, 91058, Erlangen, Germany

5 Faculty of Physics
Adam Mickiewicz University, Uniwersytetu Poznanskiego 2, Poznan 61-614, Poland

*george.fytas@mpip-mainz.mpg.de; nicolas.vogel@fau.de

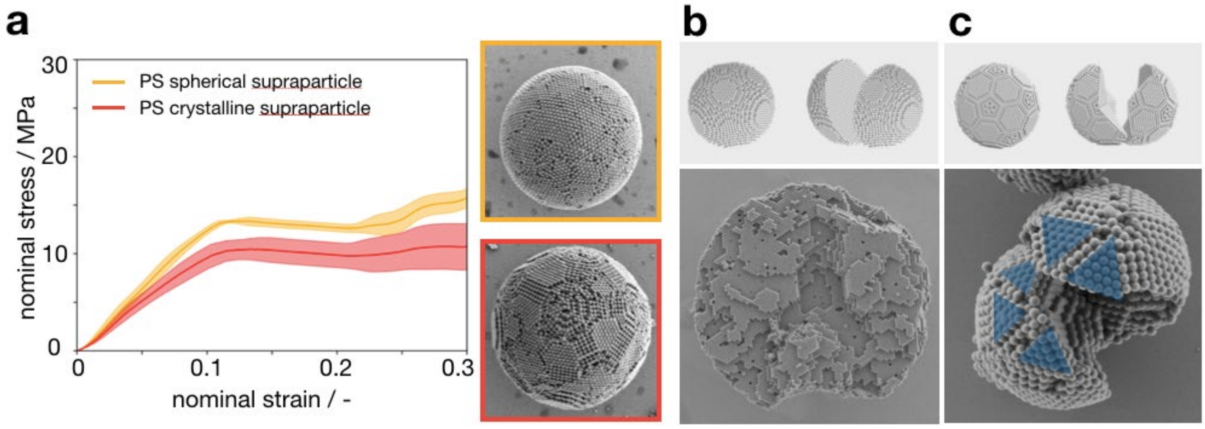

Figure S1, Comparison of crystalline and spherical supraparticles, **a**, ductile fracture of spherical disordered (yellow) and crystalline supraparticles (red) of polystyrene with nonionic PPP surfactant under compression. **b,c**, crystalline supraparticles under ultrasonication fracture at the grain boundary of (111) crystal planes.

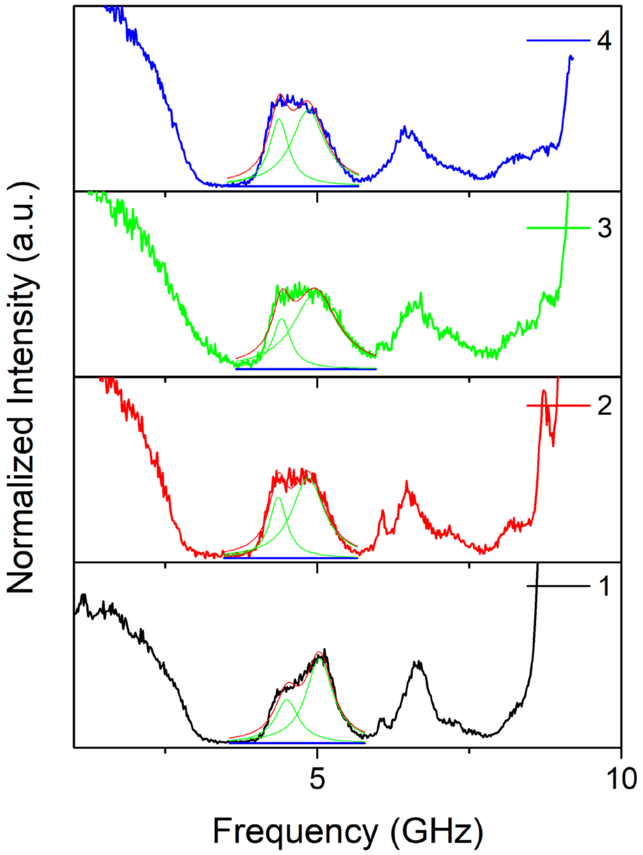

Figure S2, Representation of the (1,2) spectral shape by two Gaussians centered at f_1 and $f_2>f_1$. The frequency $f_L=2f_1-f_2$ accounts for the presence of the interparticle interactions. While the position of f_1 and f_2 and their relative intensities of the two peaks vary among the different infiltrated polystyrene opals, f_L=3.82±0.07 GHz is unaffected by the infiltrated fluids and represents the shear modulus of the PS. The numbers 1 to 4 correspond to: polystyrene colloidal crystalline film with face-center cubic structure as prepared; infiltrated with HFE oil with 0.1% nonionic PPP surfactant; infiltrated with pure HFE oil; infiltrated with HFE oil with 0.1% anionic Kyrtox surfactant.

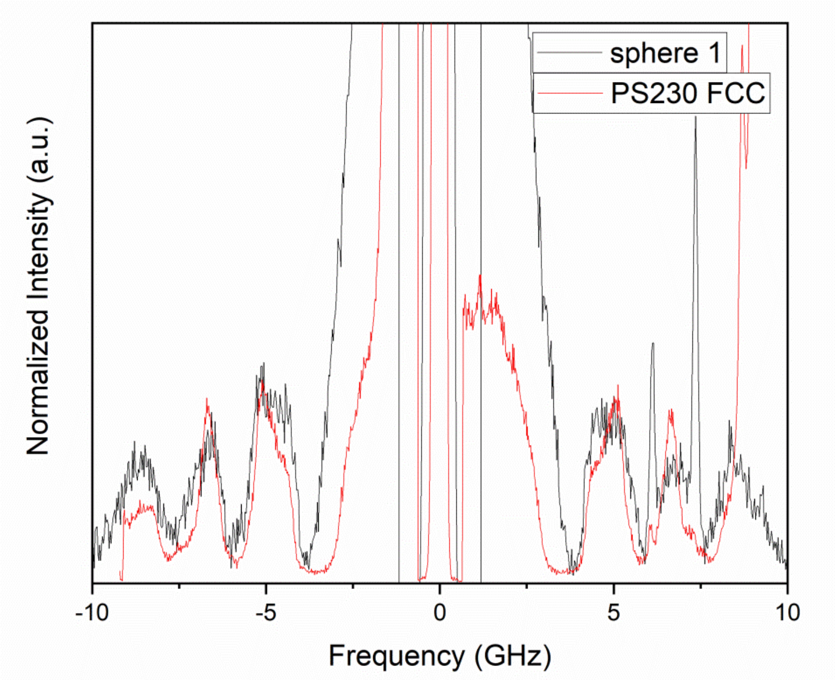

Figure S3, BLS spectrum of ordered supraparticle (Sphere 1) and crystalline film of the primary polystyrene prepared with Krytox surfactant.

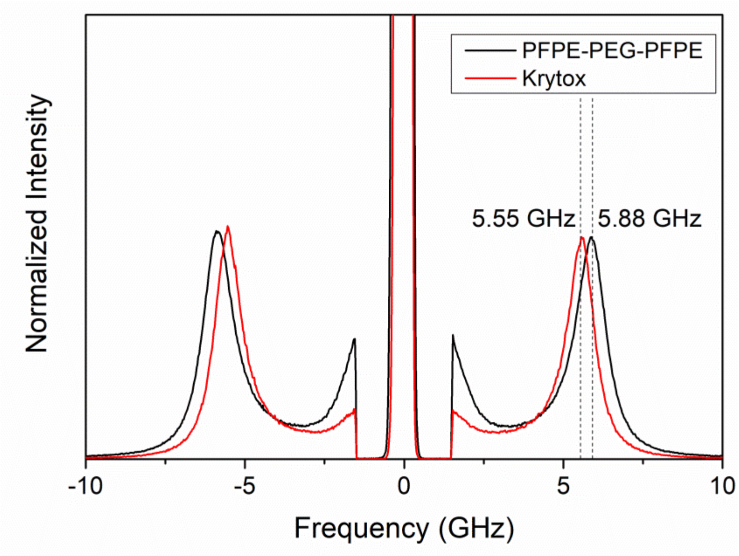

Figure S4, Backscattering spectra of the two neat PPP and Krytox surfactants. Using refractive index 1.3 for Krytox and 1.33 for PPP the sound velocity amounts to 1140 m/s and 1170 m/s, respectively.
